# The head-to-head photodimer of indeno­indene

**DOI:** 10.1107/S2414314620003077

**Published:** 2020-03-05

**Authors:** Heiner Detert, Nina Jacobs, Dieter Schollmeyer

**Affiliations:** a Johannes Gutenberg University Mainz, Department of Chemistry, Duesbergweg 10-14, 55099 Mainz, Germany; Universita degli Studi di Parma, Italy

**Keywords:** crystal structure, polycyclic hydro­carbon, strain, cyclo­butane

## Abstract

Irradiation of 1-(1-benzo­cyclo­butenyl­idene)benzo­cyclo­butene gives indeno­indene and its head-to-head photodimer nona­cyclo­[9.7.7.7^2,10^.0^1,11^.0^2,10^.0^3,8^.0^12,17^.0^19,24^.0^26,31^]dotriaconta-3,5,7,12,14,16,19,21,23,26,28,30-dodeca­ene, C_32_H_24_. The mol­ecule is built from four essentially planar indane units attached to an elongated cyclo­butane ring. In the crystal, C—H⋯π inter­actions connect mol­ecules into layers parallel to the *bc* plane.

## Abstract 2

The crystal structure of the head-to-head photodimer of indeno­indene, obtained by irradiation of 1-(1-benzo­cyclo­butenyl­idene)benzo­cyclo­butene, is reported.

## Graphical abstract

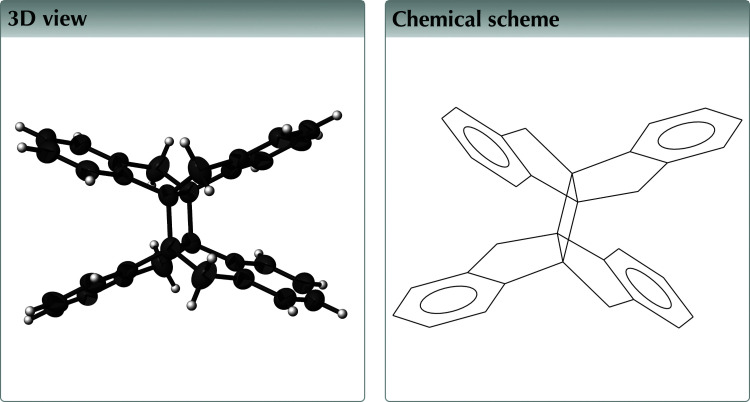

## Structure description

The photo­cyclo­addition of 5,10-di­hydro­indeno­[2,1-*a*]indene (Detert & Schollmeyer, 2019 ▸) has been studied by Shim (Shim *et al.*, 1983 ▸) and Wolff (Wolff *et al.*, 1992 ▸). Head-to-head and head-to-tail photodimers have been found in a 1: 2 ratio (Shim & Chae, 1982 ▸). As part of a project on strained (Detert *et al.*, 2009 ▸; Dobryakov *et al.*, 2016 ▸; Krohn *et al.*, 2019 ▸) and polycyclic hydro­carbons (Krämer *et al.*, 2009 ▸; Detert & Meier, 1997*a* ▸,*b* ▸), indeno­indene was prepared in a photochemical rearrangement of 1-(1-benzo­cyclo­butenyl­idene)benzo­cyclo­butene; concomitant 2 + 2-cyclo­addition of indeno­indene produced the title compound as a byproduct.

**Figure 1:**
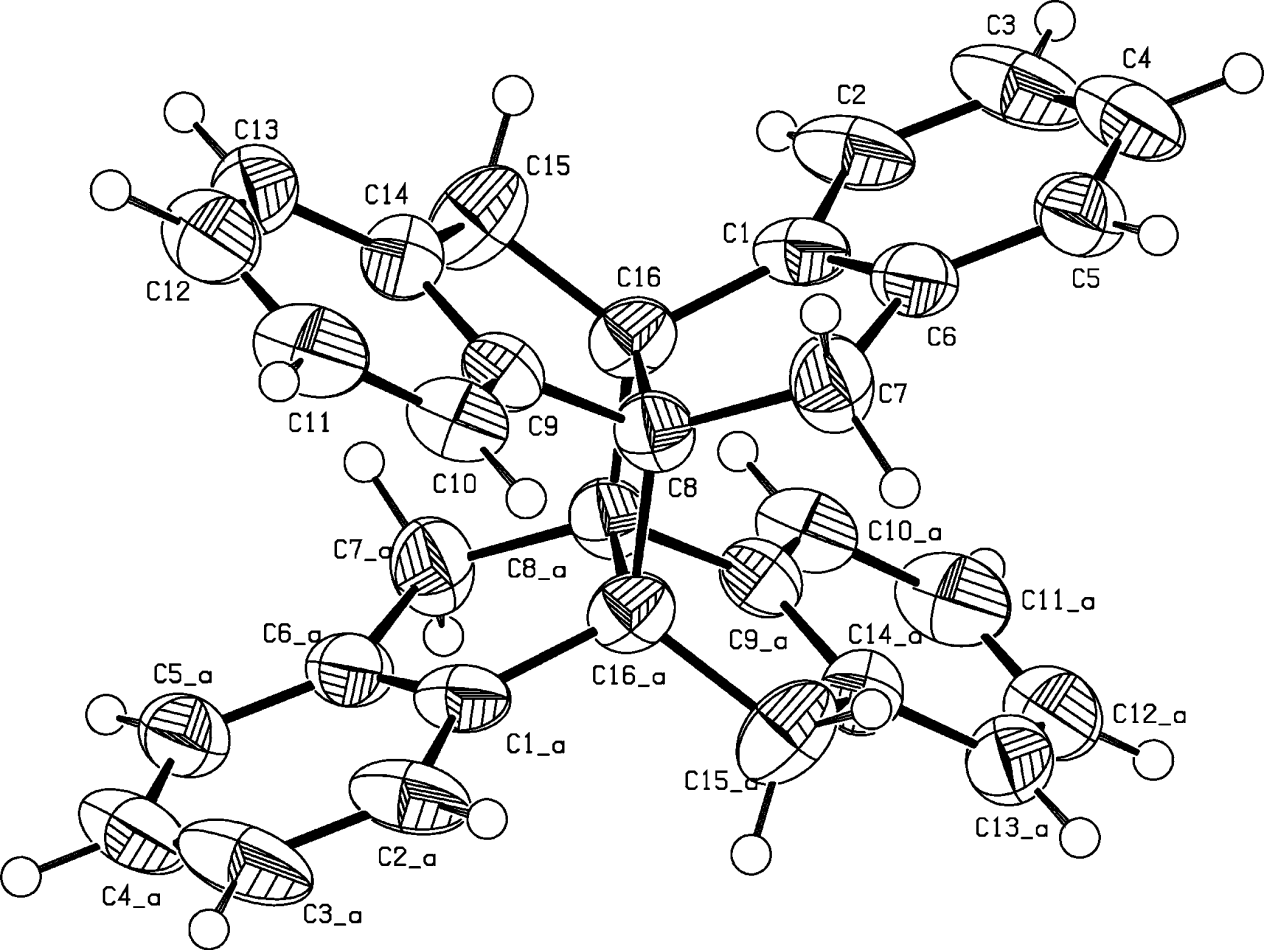
Perspective view of the title compound. Displacement ellipsoids are drawn at the 50% probability level. The second part of the mol­ecule is generated by the symmetry operation 1 − *x*, 1 − *y*, 1 − *z*.

**Figure 2:**
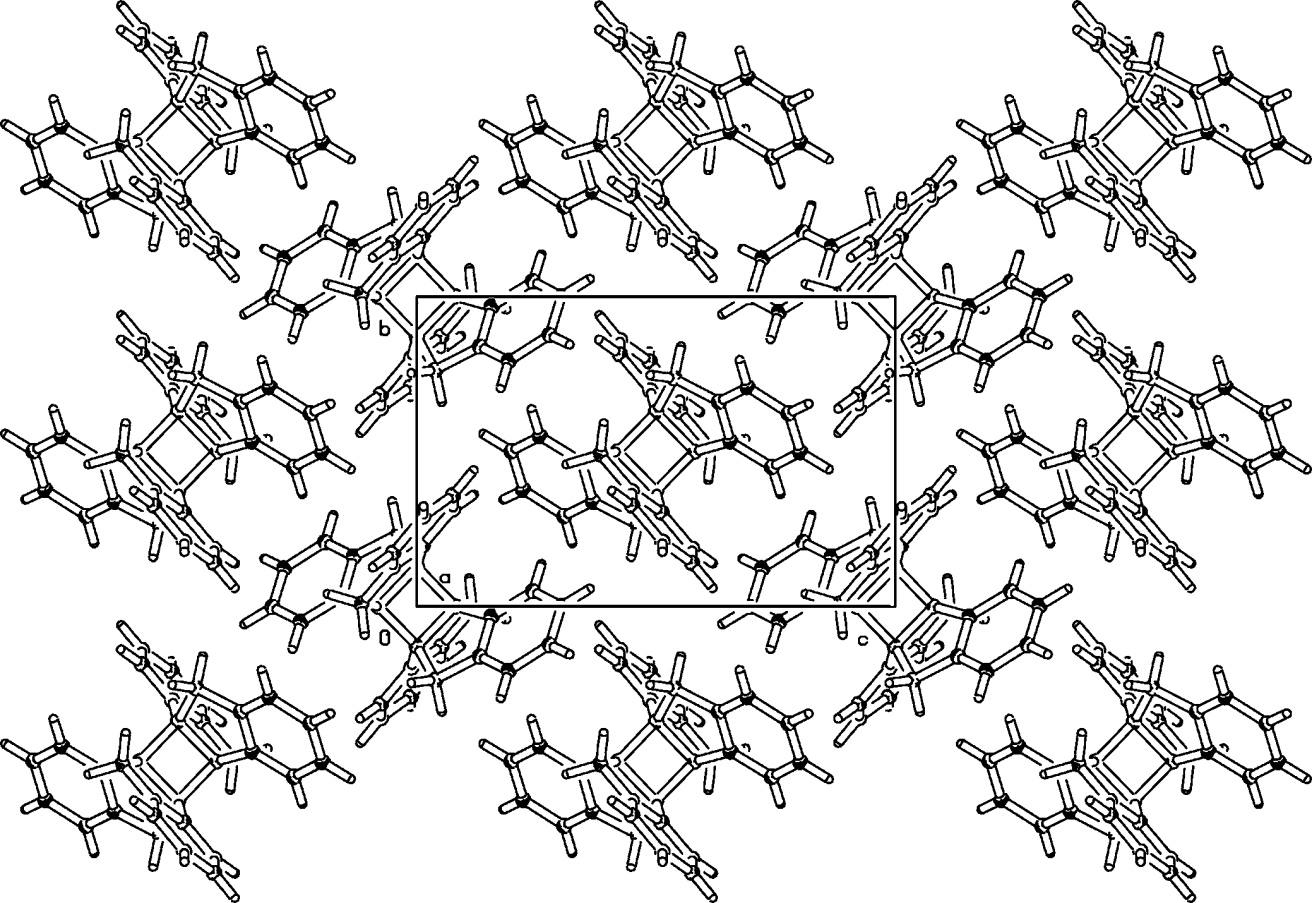
Partial packing diagram of the title compound. View along the *a* axis.

The monoclinic unit cell of the title compound (Fig. 1 ▸) contains two centrosymmetrical mol­ecules. The indane units, though containing *sp* ^3^ carbons, are essentially planar with a maximum deviation of 0.043 (2) Å at C16 from the mean plane. An angle of 51.53 (5)° is opened by the least-squares planes of indanes annulated to the cyclo­butane [C8, C16, C8^i^, C16^i^; symmetry code: (i) 1 − *x*, 1 − *y*, 1 − *z*], nearly identical to the angle of 52.28 (8)° between the planes of indanes on opposite sides of the cyclo­butane. The C—C bonds in the central cyclo­butane ring are largely elongated, the C8—C16 bond is 1.569 (3) Å long and the C8–C16^i^ bond, connecting the indanoindane units, is even more stretched to 1.597 (3) Å. This is due to the ecliptic conformation of vicinal methyl­ene groups, the minimal distance between C7—H and C15^i^—H is 1.95 Å, lower than the sum of the van der Waals radii. Bond angles in the cyclo­butane are close to orthogonal, C8—C16—C8^i^ = 90.41 (15) and C16—C8—C16^i^ = 89.59 (15)°. Bond angles on the cyclo­butane are much larger, C1—C16—C8^i^ = 115.12 (17), C1—C16—C15 = 116.65 (19)° and C15—C16—C8^i^ = 118.80 (19)°. In the crystal, mol­ecules are linked by C—H⋯π inter­actions (Fig. 2 ▸, Table 1 ▸), forming layers parallel to the *bc* plane.

## Synthesis and crystallization

Indeno­[2,1-*a*]indene (Detert & Schollmeyer, 2019 ▸) was prepared from benzo­cyclo­butenone (Schiess & Heitzmann, 1977 ▸), according to literature procedures (Detert & Schollmeyer, 2018 ▸; Oelgemöller *et al.*, 2002 ▸). The photochemical rearrangement was performed in a falling film photoreactor (Normag, Ilmenau) equipped with a medium pressure mercury lamp (TQ 718) in a diluted solution (0.1%) in petroleum ether. Contrary to the irradiation of indeno­indene in benzene (Shim & Chae, 1982 ▸), the head-to-head isomer was the main dimerization product. Crystals were obtained by slow evaporation of a petroleum ether solution.

## Refinement

Crystal data, data collection and structure refinement details are summarized in Table 2 ▸.

## Supplementary Material

Crystal structure: contains datablock(s) I, global. DOI: 10.1107/S2414314620003077/rz4037sup1.cif

Structure factors: contains datablock(s) I. DOI: 10.1107/S2414314620003077/rz4037Isup2.hkl

Supporting information file. DOI: 10.1107/S2414314620003077/rz4037Isup3.cml

CCDC reference: 1988066

Additional supporting information:  crystallographic information; 3D view; checkCIF report

## Figures and Tables

**Table 1:** Hydrogen-bond geometry (Å, °) *Cg*1 is the centroid of the C9–C14 ring.

*D*—H⋯*A*	*D*—H	H⋯*A*	*D*⋯*A*	*D*—H⋯*A*
C3—H3⋯*Cg*1^i^	0.95	2.77	3.713 (3)	171

Symmetry code: (i) 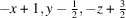 .

**Table 2:** Experimental details

Crystal data	
Chemical formula	C_32_H_24_
*M*_r_	408.51
Crystal system, space group	Monoclinic, *P*2_1_/*c*
Temperature (K)	193
*a*, *b*, *c* (Å)	9.3106 (11), 8.7232 (8), 13.4776 (12)
β (°)	91.959 (8)
*V* (Å^3^)	1093.99 (19)
*Z*	2
Radiation type	Mo *K*α
μ (mm^−1^)	0.07
Crystal size (mm)	0.40 × 0.32 × 0.17

Data collection	
Diffractometer	Stoe *IPDS* 2T
No. of measured, independent and observed [*I* > 2σ(*I*)] reflections	5905, 2604, 1416
*R*_int_	0.051
(sin θ/λ)_max_ (Å^−1^)	0.661

Refinement	
*R*[*F* ^2^ > 2σ(*F* ^2^)], *wR*(*F* ^2^), *S*	0.061, 0.196, 0.97
No. of reflections	2604
No. of parameters	145
H-atom treatment	H-atom parameters constrained
Δρ_max_, Δρ_min_ (e Å^−3^)	0.19, −0.22

Computer programs: *X-AREA* and *X-RED* (Stoe & Cie, 1996 ▸), *SIR2004* (Burla *et al.*, 2005 ▸), *SHELXL2018/3* (Sheldrick, 2015 ▸) and *PLATON* (Spek, 2020 ▸).

## full crystallographic data

### Crystal data

**Table:**

C_32_H_24_	*F*(000) = 432
*M**_r_* = 408.51	*D*_x_ = 1.240 Mg m^−^^3^
Monoclinic, *P*2_1_/*c*	Mo *K*α radiation, λ = 0.71073 Å
*a* = 9.3106 (11) Å	Cell parameters from 4760 reflections
*b* = 8.7232 (8) Å	θ = 2.8–28.4°
*c* = 13.4776 (12) Å	µ = 0.07 mm^−^^1^
β = 91.959 (8)°	*T* = 193 K
*V* = 1093.99 (19) Å^3^	Block, colourless
*Z* = 2	0.40 × 0.32 × 0.17 mm

### Data collection

**Table:**

Stoe IPDS 2T diffractometer	1416 reflections with *I* > 2σ(*I*)
Radiation source: sealed X-ray tube, 12 x 0.4 mm long-fine focus	*R*_int_ = 0.051
Detector resolution: 6.67 pixels mm^-1^	θ_max_ = 28.0°, θ_min_ = 2.8°
rotation method scans	*h* = −12→12
5905 measured reflections	*k* = −11→11
2604 independent reflections	*l* = −17→16

### Refinement

**Table:**

Refinement on *F*^2^	Primary atom site location: structure-invariant direct methods
Least-squares matrix: full	Hydrogen site location: inferred from neighbouring sites
*R*[*F*^2^ > 2σ(*F*^2^)] = 0.061	H-atom parameters constrained
*wR*(*F*^2^) = 0.196	*w* = 1/[σ^2^(*F*_o_^2^) + (0.1156*P*)^2^] where *P* = (*F*_o_^2^ + 2*F*_c_^2^)/3
*S* = 0.97	(Δ/σ)_max_ < 0.001
2604 reflections	Δρ_max_ = 0.19 e Å^−^^3^
145 parameters	Δρ_min_ = −0.22 e Å^−^^3^
0 restraints	

### Special details

**Table:**

Geometry. All esds (except the esd in the dihedral angle between two l.s. planes) are estimated using the full covariance matrix. The cell esds are taken into account individually in the estimation of esds in distances, angles and torsion angles; correlations between esds in cell parameters are only used when they are defined by crystal symmetry. An approximate (isotropic) treatment of cell esds is used for estimating esds involving l.s. planes.
Refinement. Hydrogen atoms attached to carbons were placed at calculated positions and were refined in the riding-model approximation with C–H = 0.95–0.99 Å, and with *U*_iso_(H) = 1.2 *U*_eq_(C).

### Fractional atomic coordinates and isotropic or equivalent isotropic displacement parameters (Å^2^)

**Table:**

	*x*	*y*	*z*	*U*_iso_*/*U*_eq_	
C1	0.4266 (2)	0.5319 (2)	0.65609 (14)	0.0434 (5)	
C2	0.3996 (3)	0.4508 (3)	0.74264 (16)	0.0586 (7)	
H2	0.455901	0.363472	0.760360	0.070*	
C3	0.2908 (4)	0.4981 (3)	0.80229 (18)	0.0738 (9)	
H3	0.271851	0.442967	0.861199	0.089*	
C4	0.2099 (3)	0.6238 (4)	0.77730 (18)	0.0675 (8)	
H4	0.134797	0.654571	0.818923	0.081*	
C5	0.2357 (3)	0.7075 (3)	0.69200 (18)	0.0566 (7)	
H5	0.179057	0.794881	0.675228	0.068*	
C6	0.3453 (2)	0.6613 (3)	0.63188 (15)	0.0450 (5)	
C7	0.3945 (3)	0.7366 (3)	0.53776 (17)	0.0553 (6)	
H7A	0.435041	0.839524	0.551922	0.066*	
H7B	0.313791	0.746705	0.488456	0.066*	
C8	0.5100 (2)	0.6283 (2)	0.49985 (14)	0.0399 (5)	
C9	0.6561 (2)	0.6969 (2)	0.48827 (14)	0.0384 (5)	
C10	0.6955 (3)	0.8137 (2)	0.42436 (16)	0.0490 (6)	
H10	0.625938	0.861017	0.381202	0.059*	
C11	0.8370 (3)	0.8597 (3)	0.42464 (19)	0.0635 (7)	
H11	0.865113	0.939744	0.381628	0.076*	
C12	0.9381 (3)	0.7905 (3)	0.48689 (19)	0.0618 (7)	
H12	1.035286	0.823562	0.486287	0.074*	
C13	0.9004 (3)	0.6743 (3)	0.54978 (18)	0.0521 (6)	
H13	0.970946	0.626587	0.591988	0.063*	
C14	0.7580 (2)	0.6277 (2)	0.55081 (16)	0.0429 (5)	
C15	0.6931 (3)	0.5091 (3)	0.6165 (2)	0.0581 (7)	
H15A	0.700371	0.541016	0.686992	0.070*	
H15B	0.741669	0.408951	0.609403	0.070*	
C16	0.5354 (2)	0.4997 (2)	0.57990 (15)	0.0419 (5)	

### Atomic displacement parameters (Å^2^)

**Table:**

	*U*^11^	*U*^22^	*U*^33^	*U*^12^	*U*^13^	*U*^23^
C1	0.0536 (13)	0.0476 (12)	0.0287 (10)	−0.0135 (10)	−0.0022 (8)	0.0010 (9)
C2	0.091 (2)	0.0540 (14)	0.0302 (11)	−0.0209 (13)	0.0020 (11)	0.0007 (10)
C3	0.120 (3)	0.0679 (18)	0.0344 (12)	−0.0366 (18)	0.0200 (14)	−0.0072 (13)
C4	0.081 (2)	0.0788 (19)	0.0440 (13)	−0.0325 (16)	0.0233 (13)	−0.0229 (13)
C5	0.0526 (15)	0.0684 (16)	0.0492 (13)	−0.0105 (12)	0.0069 (11)	−0.0150 (12)
C6	0.0465 (13)	0.0534 (13)	0.0350 (10)	−0.0082 (10)	0.0017 (9)	−0.0040 (9)
C7	0.0527 (15)	0.0652 (15)	0.0483 (12)	0.0166 (12)	0.0070 (10)	0.0127 (11)
C8	0.0440 (12)	0.0416 (11)	0.0342 (10)	0.0045 (9)	0.0033 (8)	0.0083 (9)
C9	0.0483 (13)	0.0309 (10)	0.0363 (10)	0.0003 (9)	0.0061 (8)	−0.0011 (8)
C10	0.0686 (17)	0.0380 (11)	0.0406 (11)	−0.0071 (11)	0.0058 (10)	0.0011 (9)
C11	0.082 (2)	0.0577 (15)	0.0518 (14)	−0.0284 (14)	0.0125 (13)	−0.0015 (12)
C12	0.0597 (17)	0.0669 (16)	0.0597 (15)	−0.0245 (13)	0.0148 (12)	−0.0135 (12)
C13	0.0468 (14)	0.0509 (13)	0.0587 (14)	−0.0052 (11)	0.0030 (11)	−0.0090 (11)
C14	0.0435 (13)	0.0358 (11)	0.0494 (12)	−0.0013 (9)	0.0017 (9)	−0.0015 (9)
C15	0.0492 (14)	0.0524 (14)	0.0719 (16)	−0.0063 (12)	−0.0124 (12)	0.0219 (12)
C16	0.0439 (12)	0.0439 (11)	0.0377 (11)	−0.0032 (9)	−0.0024 (9)	0.0117 (9)

### Geometric parameters (Å, º)

**Table:**

C1—C6	1.392 (3)	C8—C16	1.569 (3)
C1—C2	1.395 (3)	C8—C16^i^	1.597 (3)
C1—C16	1.494 (3)	C9—C14	1.386 (3)
C2—C3	1.379 (4)	C9—C10	1.392 (3)
C2—H2	0.9500	C10—C11	1.376 (4)
C3—C4	1.366 (4)	C10—H10	0.9500
C3—H3	0.9500	C11—C12	1.379 (4)
C4—C5	1.389 (4)	C11—H11	0.9500
C4—H4	0.9500	C12—C13	1.374 (3)
C5—C6	1.384 (3)	C12—H12	0.9500
C5—H5	0.9500	C13—C14	1.388 (3)
C6—C7	1.513 (3)	C13—H13	0.9500
C7—C8	1.532 (3)	C14—C15	1.501 (3)
C7—H7A	0.9900	C15—C16	1.535 (3)
C7—H7B	0.9900	C15—H15A	0.9900
C8—C9	1.500 (3)	C15—H15B	0.9900
			
C6—C1—C2	119.7 (2)	C14—C9—C10	120.4 (2)
C6—C1—C16	111.58 (18)	C14—C9—C8	111.53 (17)
C2—C1—C16	128.7 (2)	C10—C9—C8	128.0 (2)
C3—C2—C1	119.5 (3)	C11—C10—C9	119.0 (2)
C3—C2—H2	120.3	C11—C10—H10	120.5
C1—C2—H2	120.3	C9—C10—H10	120.5
C4—C3—C2	120.5 (2)	C10—C11—C12	120.5 (2)
C4—C3—H3	119.8	C10—C11—H11	119.8
C2—C3—H3	119.8	C12—C11—H11	119.8
C3—C4—C5	121.1 (3)	C13—C12—C11	121.0 (2)
C3—C4—H4	119.5	C13—C12—H12	119.5
C5—C4—H4	119.5	C11—C12—H12	119.5
C6—C5—C4	118.9 (3)	C12—C13—C14	119.1 (2)
C6—C5—H5	120.5	C12—C13—H13	120.4
C4—C5—H5	120.5	C14—C13—H13	120.4
C5—C6—C1	120.3 (2)	C9—C14—C13	120.0 (2)
C5—C6—C7	128.0 (2)	C9—C14—C15	112.24 (19)
C1—C6—C7	111.7 (2)	C13—C14—C15	127.7 (2)
C6—C7—C8	104.37 (18)	C14—C15—C16	104.30 (17)
C6—C7—H7A	110.9	C14—C15—H15A	110.9
C8—C7—H7A	110.9	C16—C15—H15A	110.9
C6—C7—H7B	110.9	C14—C15—H15B	110.9
C8—C7—H7B	110.9	C16—C15—H15B	110.9
H7A—C7—H7B	108.9	H15A—C15—H15B	108.9
C9—C8—C7	115.99 (18)	C1—C16—C15	115.65 (19)
C9—C8—C16	103.94 (16)	C1—C16—C8	104.37 (17)
C7—C8—C16	107.62 (17)	C15—C16—C8	107.63 (17)
C9—C8—C16^i^	115.41 (17)	C1—C16—C8^i^	115.12 (17)
C7—C8—C16^i^	118.91 (19)	C15—C16—C8^i^	118.80 (19)
C16—C8—C16^i^	89.59 (15)	C8—C16—C8^i^	90.41 (15)
			
C6—C1—C2—C3	1.2 (3)	C10—C9—C14—C13	−0.2 (3)
C16—C1—C2—C3	−178.4 (2)	C8—C9—C14—C13	179.10 (18)
C1—C2—C3—C4	−0.2 (4)	C10—C9—C14—C15	177.7 (2)
C2—C3—C4—C5	−0.5 (4)	C8—C9—C14—C15	−3.0 (3)
C3—C4—C5—C6	0.1 (4)	C12—C13—C14—C9	0.6 (3)
C4—C5—C6—C1	0.9 (3)	C12—C13—C14—C15	−176.9 (2)
C4—C5—C6—C7	−178.3 (2)	C9—C14—C15—C16	5.6 (3)
C2—C1—C6—C5	−1.6 (3)	C13—C14—C15—C16	−176.7 (2)
C16—C1—C6—C5	178.12 (19)	C6—C1—C16—C15	116.9 (2)
C2—C1—C6—C7	177.8 (2)	C2—C1—C16—C15	−63.4 (3)
C16—C1—C6—C7	−2.5 (3)	C6—C1—C16—C8	−1.1 (2)
C5—C6—C7—C8	−175.6 (2)	C2—C1—C16—C8	178.6 (2)
C1—C6—C7—C8	5.1 (3)	C6—C1—C16—C8^i^	−98.5 (2)
C6—C7—C8—C9	−121.4 (2)	C2—C1—C16—C8^i^	81.2 (3)
C6—C7—C8—C16	−5.5 (2)	C14—C15—C16—C1	−122.1 (2)
C6—C7—C8—C16^i^	94.1 (2)	C14—C15—C16—C8	−6.0 (2)
C7—C8—C9—C14	116.93 (19)	C14—C15—C16—C8^i^	94.6 (2)
C16—C8—C9—C14	−1.0 (2)	C9—C8—C16—C1	127.77 (17)
C16^i^—C8—C9—C14	−97.3 (2)	C7—C8—C16—C1	4.2 (2)
C7—C8—C9—C10	−63.9 (3)	C16^i^—C8—C16—C1	−116.11 (18)
C16—C8—C9—C10	178.2 (2)	C9—C8—C16—C15	4.4 (2)
C16^i^—C8—C9—C10	81.9 (3)	C7—C8—C16—C15	−119.2 (2)
C14—C9—C10—C11	−0.3 (3)	C16^i^—C8—C16—C15	120.5 (2)
C8—C9—C10—C11	−179.5 (2)	C9—C8—C16—C8^i^	−116.13 (18)
C9—C10—C11—C12	0.4 (4)	C7—C8—C16—C8^i^	120.3 (2)
C10—C11—C12—C13	0.1 (4)	C16^i^—C8—C16—C8^i^	−0.002 (1)
C11—C12—C13—C14	−0.6 (4)		

Symmetry code: (i) −*x*+1, −*y*+1, −*z*+1.

### Hydrogen-bond geometry (Å, º)

*Cg*1 is the centroid of the C9–C14 ring.

**Table:**

*D*—H···*A*	*D*—H	H···*A*	*D*···*A*	*D*—H···*A*
C3—H3···*Cg*1^ii^	0.95	2.77	3.713 (3)	171

Symmetry code: (ii) −*x*+1, *y*−1/2, −*z*+3/2.
